# MicroRNA expression distinguishes SCLC from NSCLC lung tumor cells and suggests a possible pathological relationship between SCLCs and NSCLCs

**DOI:** 10.1186/1756-9966-29-75

**Published:** 2010-06-17

**Authors:** Liqin Du, Jeoffrey J Schageman, Irnov, Luc Girard, Scott M Hammond, John D Minna, Adi F Gazdar, Alexander Pertsemlidis

**Affiliations:** 1 Simmons Comprehensive Cancer Center, UT Southwestern Medical Center, Dallas, Texas, USA; 2 McDermott Center for Human Growth and Development, UT Southwestern Medical Center, Dallas, Texas, USA; 3 Division of Basic Sciences, Southwestern Graduate School of Biomedical Sciences, UT Southwestern Medical Center, Dallas, Texas, USA; 4 Hamon Center for Therapeutic Oncology Research, UT Southwestern Medical Center, Dallas, Texas, USA; 5 Department of Pharmacology, UT Southwestern Medical Center, Dallas, Texas, USA; 6 Department of Internal Medicine, UT Southwestern Medical Center, Dallas, Texas, USA; 7 Department of Pathology, UT Southwestern Medical Center, Dallas, Texas, USA; 8 Lineberger Comprehensive Cancer Center, School of Medicine, University of North Carolina School at Chapel Hill, Chapel Hill, North Carolina, USA

## Abstract

**Background:**

Recent studies have shown that microRNAs (miRNAs) play roles in tumorigenesis and are reliable classifiers of certain cancer types and subtypes. However, the role of miRNAs in the pathogenesis and diagnosis of small cell carcinoma (SCLC), the majority of which represent the most aggressive lung tumors, has not been investigated.

**Methods:**

In order to explore miRNA involvement in the pathogenesis of small cell lung carcinoma (SCLC) and the potential role of miRNAs in SCLC diagnosis, we compared the miRNA expression profile of a set of SCLC cell lines to that of a set of non-small cell lung cancer (NSCLC) cell lines and normal immortalized human bronchial epithelial cells (HBECs) using microarray analysis.

**Results:**

Our results show that miRNA profiles reliably distinguish SCLC cell lines from NSCLC and HBEC cell lines. Further analysis of the miRNA expression profile of the two subtypes of lung cancer cell lines indicates that the expression levels of the majority of the miRNAs that are differentially expressed in SCLC cells relative to NSCLC cells and HBECs show a progressive trend from HBECs to NSCLC cells to SCLC cells.

**Conclusions:**

The distinctive miRNA expression signature of SCLCs relative to NSCLCs and HBECs suggests that miRNA profiles have the potential to serve as a diagnostic marker of SCLC lung tumors. The progressive trend of miRNA profile changes from HBECs to NSCLCs to SCLCs suggests a possible pathological relationship between SCLCs and NSCLCs, and suggests that the increasing dysregulation of miRNA expression may play a role in lung tumor progression. The specific role of these miRNAs in lung tumor pathogenesis and differentiation need to be investigated further in future studies.

## Background

Small cell lung carcinoma (SCLC) is the most aggressive subtype of all lung tumors [1]. The poor survival rate of patients with SCLC is largely due to late detection and the lack of therapeutic regimens specifically targeted to SCLC [2,3]; thus, therapeutic improvement depends on a better understanding of the mechanisms underlying SCLC tumorigenesis and developing targeted therapy for this class of lung cancers. Although decades of work have led to better understanding of the genetic abnormalities in SCLC [1,4], these still cannot completely explain the aggressive phenotype that distinguishes it from other lung cancer subtypes. There is clearly an urgent need for continued efforts to understand SCLC tumorigenesis and to identify early diagnostic markers and therapeutic targets for SCLC.

A recently discovered class of small noncoding RNAs, microRNAs (miRNAs), regulates gene expression primarily by binding to sequences in the 3' untranslated region (3'UTR) of expressed mRNAs, resulting in decreased protein expression either by repression of translation or by enhancement of mRNA degradation. miRNAs have been shown to have a variety of regulatory functions and to play roles in controlling cancer initiation and progression [5]. Many studies have demonstrated dysregulation of particular miRNAs in various cancer types and investigated the mechanisms of specific miRNAs in tumorigenesis [5-7]. In the context of lung cancer, several studies have attempted to distinguish the miRNA profiles of histological subtypes showing the potential of miRNA profiles as diagnostic markers for distinguishing specific subtypes, such as squamous cell carcinoma and adenocarcinoma [8,9]. Moreover, tumor suppressor genes and oncogenes that play crucial roles in lung tumorigenesis have been demonstrated to be targets of miRNAs [10-12], and manipulation of miRNA levels has been used to control lung cancer cell survival and proliferation *in vitro *and *in vivo *[13-16]. Few studies, however, have focused on the role of miRNAs in the pathogenesis of SCLC [17]. Primary tissue specimens are difficult to obtain as most SCLC tumors are not surgically resected [4,18], underscoring the importance of cell lines for studying this disease [19,20]. In order to characterize the expression of miRNAs in SCLC and explore the potential role of miRNAs in SCLC tumorigenesis, we profiled and compared the expression levels of a group of miRNAs in a set of lung cancer cell lines, including SCLC and non-small cell lung cancer (NSCLC) cell lines and immortalized human bronchial epithelial cells (HBECs).

## Materials and methods

### Cell lines

19 cell lines (Table 1), including 16 lung cancer cell lines [21], and 3 HBEC cell lines immortalized via ectopic expression of *cdk4 *and *hTERT *[22], were obtained from the Hamon Center for Therapeutic Oncology Research at UT Southwestern Medical Center. All cancer cell lines were grown in RPMI-1640 medium (Sigma, St. Louis, MO) supplemented with 5% fetal bovine serum. HBECs were grown in KSFM medium supplemented with bovine pituitary extract and recombinant human epidermal growth factor (Gibco, Carlsbad, CA). All cell lines were grown in a humidified atmosphere with 5% CO_2 _at 37°C.

**Table 1:** Histological classification of the lung cancer cell lines

Cell Line	Tumor Subtype	Age	Ethnicity	Gender	Source	Site
NCI-H146	SCLC	59	Caucasian	M	metastasis	bone
NCI-H187	SCLC	47	Caucasian	M	metastasis	pleural
NCI-H209	SCLC	55	Caucasian	M	metastasis	bone
NCI-H526	SCLC	55	Caucasian	M	metastasis	bone
NCI-H889	SCLC	69	Caucasian	F	metastasis	lymph
NCI-H1672	SCLC	58	Caucasian	M	primary	lung
NCI-H2107	SCLC	36	Black	M	metastasis	bone
NCI-H2171	SCLC	50	Caucasian	M	metastasis	pleural
NCI-H2195	SCLC	67	Caucasian	M	metastasis	bone
NCI-H157	NSCLC (squamous)	59	Caucasian	M	metastasis	pleural
NCI-H1819	NSCLC (adenocarcinoma)	55	Caucasian	F	metastasis	lymph
NCI-H2052	NSCLC (mesothelioma)	65	Caucasian	M	metastasis	pleural
NCI-H2887	NSCLC (squamous)	31	unknown	M	primary	lung
HCC366	NSCLC (adenosquamous)	80	unknown	F	primary	lung
HCC1195	NSCLC (adenocarcinoma)	47	Black	M	primary	lung
HCC2450	NSCLC (squamous)	52	Caucasian	M	primary	lung
HBEC2-KT	Immortalized Normal	68		M	NA	lung
HBEC3-KT	Immortalized Normal	65		F	NA	lung
HBEC4-KT	Immortalized Normal	71		F	NA	lung

The lung cancer cell lines were established from tissue specimens obtained from lung cancer patients [73]. The subtype of each lung cancer cell line is based on histological examination of the tumor from which the line was derived. Patient age, ethnicity, and gender are listed along with the source of the tissue sample and the site from which the sample was derived.

### RNA isolation and miRNA microarray

Total RNA was extracted using TRIzol (Invitrogen, Carlsbad, CA), and labeled with a fluorescent modified dinucleotide (5'-phosphate-cytidyl-uridyl-Cy3-3') using T4 RNA ligase, according to Thomson [23]. Oligonucleotide probes antisense to the published mature sequences for 136 conserved human miRNAs were synthesized and spotted in duplicate on Corning GAPS-2 coated slides using a robotic spotter [23]. Samples were hybridized to the array, along with an equimolar reference oligonucleotide set corresponding to the 136 mature microRNAs, which had been labeled with Cy5. Array images were obtained and analyzed with a GenePix 4000A scanner and GenePix Pro 4.1 software (Axon Instruments). We measured raw signal at 532 nm (Cy3) and 635 nm (Cy5), and the expression level of each miRNA was calculated as the ratio of the intensities for each sample to the reference. We then calculated the relative expression of each miRNA in each cell line by normalizing to the overall signal observed for each cell line measurement, and averaged duplicate spots and replicate cell line measurements.

### Hierarchical clustering analysis

The miRNA expression data was log-transformed, normalized by median centering, and then clustered using the Cluster and TreeView software packages [24]. The entire dataset was clustered both on cell lines and on miRNAs using average linkage hierarchical clustering based on Pearson correlation.

### Linear discriminant analysis

We defined three groups of cell lines based on annotated histology of the tumor from which the cell line was derived {SCLC, NSCLC and HBEC}. Each cell line can be considered a point in the multi-dimensional space defined by the miRNA expression. Given the assignment of the cell lines into the three groups, we applied linear discriminant analysis (LDA, using the "lda" function as implemented in the R package MASS) [25,26], which attempts to maximize the ratio of between-group variance to within-group variance of the dataset. The result is a linear combination of features that characterize or separate the groups and can be used to reduce the dimensionality of the data and to visualize the relationships between the groups in expression space.

### Statistical analysis

The significance of differential expression of individual miRNAs between the groups was determined by two-tailed unpaired t-test, correcting for multiple comparisons using the Benjamini-Hochberg false discovery rate (FDR) method [27]. The trend in expression of each miRNA across the three groups of cell lines was tested using the Jonckheere-Terpstra test, a non-parametric test for ordered differences among groups [28]. It is designed to detect alternatives of ordered group differences with expression of an individual miRNA increasing or decreasing monotonically across the three ordered groups (SCLCs, NSCLCs and HBECs), which can be expressed as μ_SCLC _≤ μ_NSCLC _≤ μ_HBEC _(or μ_SCLC _≥ μ_NSCLC _≥ μ_HBEC_), with at least one of the inequalities being strict, where μ_i _denotes the mean expression of a given miRNA in group i.

## Results

### Hierarchical clustering classifies cell lines as distinct groups that are consistent with their histological classification

**Figure 1:**
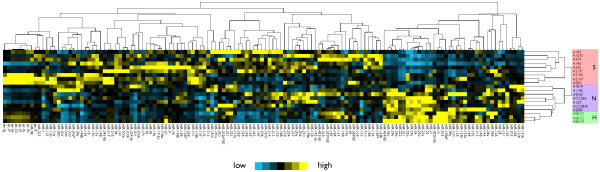
**Clustering of cell lines by miRNA expression distinguishes SCLC cell lines from NSCLCs and normal HBECs**. Shown is a heatmap representation of the expression of 136 miRNAs in 19 cell lines, with blue indicating relative under-expression and yellow indicating relative over-expression. (S, SCLC; N, NSCLC; H, HBEC).

In order to examine whether miRNA expression is informative in distinguishing SCLC cells from NSCLC cells as well as normal lung cells, we measured the expression levels of 136 miRNAs in a panel of cell lines by miRNA microarray. The panel comprised three groups of cell lines that were derived from human lung tumors or normal human lung tissue, including 9 SCLC cell lines, 7 NSCLC cell lines and 3 HBEC lines (Table 1). After normalization, we clustered the miRNA expression data using unsupervised clustering. As shown in Figure 1, the overall expression profile divides the cell lines into two large groups, recapitulating the histological classification of the cell lines and separating the group of SCLC cell lines from the group of NSCLC and HBEC cell lines. This indicates that the SCLC cell lines have a distinct expression profile from that of NSCLCs and normal HBECs. In addition, the NSCLCs cluster separately from the HBECs, indicating that expression of specific miRNAs can also classify NSCLCs from HBECs, which is consistent with a previous report [29].

### Specific miRNAs are expressed at significantly different levels between the lung cancer cell lines and HBECs as well as between the lung cancer subtypes

While the overall miRNA expression profile clusters the cell lines into groups that are consistent with histological features, the determinants of that clustering are individual miRNAs that are differentially expressed between the groups. Such differentially expressed miRNAs have the potential to serve as diagnostic markers of lung cancer as well as of specific histological subtypes. In order to identify microRNAs with significant differential expression in lung cancer cells relative to HBECs as well as between lung cancer cell subtypes, we divided the set of cell lines into three groups according to the histological classification of the cell lines and the hierarchical clustering results: SCLC (9 samples), NSCLC (7 samples) and HBEC (3 samples), and assessed differential expression of individual miRNAs between the groups by t-test.

**Figure 2:**
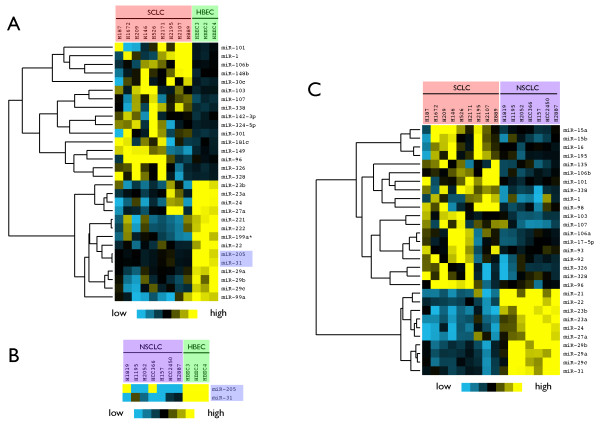
**Specific miRNAs are differentially expressed between SCLC, NSCLC and HBEC cell lines**. We divided the cell lines into three groups: SCLC (9 samples), NSCLC (7 samples), and HBECs, compared the groups pairwise, and assessed the significance of differential expression of each miRNA. **(A) **miRNAs that are differentially expressed between SCLCs and HBECs. **(B) **miRNAs that are differentially expressed between NSCLCs and HBECs. **(C) **miRNAs that are differentially expressed between SCLCs and NSCLCs. Yellow indicates relative over-expression, and blue indicates relative under-expression.

Our results identified more miRNAs as classifying SCLC cells from HBECs than as classifying NSCLC cells from HBECs. As shown in Figure 2A, 30 miRNAs were significantly differentially expressed between the SCLC and HBEC cell lines at an FDR-corrected threshold of 0.05, with 16 miRNAs over-expressed and 14 miRNAs under-expressed in SCLC compared to HBECs. Only two miRNAs (miR-31 and miR-205) were significantly differentially expressed between the NSCLC and HBEC cell lines, as shown in Figure 2B. The comparison between SCLC and NSCLC cell lines is shown in Figure 2C. 29 miRNAs were significantly differentially expressed between the SCLC and NSCLC cell lines, of which 19 are over-expressed in SCLC cell lines relative to NSCLCs and 10 are under-expressed. The miRNAs that are identified as differentially expressed between SCLC cells and NSCLC cells may serve as diagnostic markers for distinguishing SCLC from NSCLC lung tumors.

### The miRNA expression profile changes progressively from normal cells to NSCLC to SCLC cells

**Figure 3:**
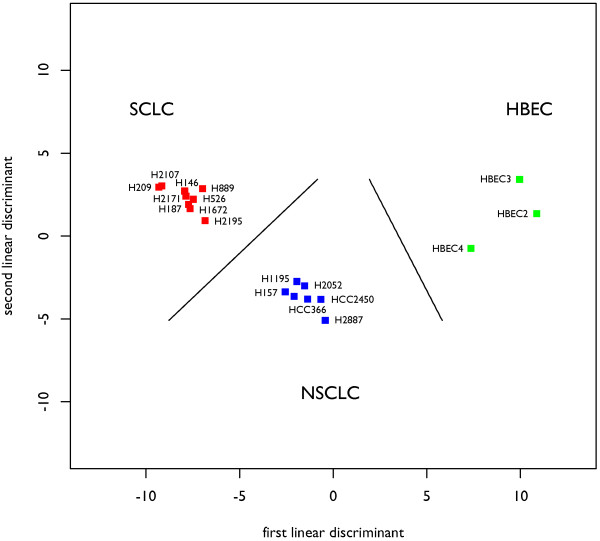
**A sequential change in expression profile from normal cells to NSCLC cells to SCLC cells**. Linear discriminant analysis places the NSCLCs between SCLCs and HBECs, suggesting a progression from HBECs to NSCLC to SCLC cell lines. The plot is a projection of the multi-dimensional space into two dimensions described by two linear discriminants, in which the individual points represent the cell lines and the classifiers are indicated by black lines. 88% of the between-class variance is explained by the first discriminant function (displayed along the x-axis of the plot).

Interestingly, the above analysis indicates that more miRNAs are differentially expressed between SCLC cell lines and HBECs than between NSCLC cell lines and HBECs. In addition, the two miRNAs that are significantly differentially expressed in NSCLCs relative to HBECs are included in the group of 30 miRNAs identified as differentially expressed in SCLCs relative to HBECS, as shown in Figure 2A. This suggests a possible pathological relationship between the three groups of cell lines. To examine this relationship, we applied linear discriminant analysis to the three groups of cell lines based on the 41 miRNAs that are identified as significantly differentially expressed as shown in Figure 2. As shown in Figure 3, 88% of the between-group variance is explained by the first discriminant function with miRNA expression placing NSCLCs between HBECs and SCLC lung tumor cells, suggesting a progressive change in expression from HBECs to NSCLC to SCLC cell lines.

To examine this relationship at the level of individual microRNAs, we applied the Jonckheere-Terpstra test for ordered means to the expression levels of each miRNA in the three groups. This allowed us to assess whether or not the expression trend followed the order {SCLC, NSCLC, HBEC}. As shown in Table 2, of the 26 miRNAs that are over-expressed in SCLC cell lines relative to non-SCLC cell lines, all 26 (100%) show ordered expression at a significance level of 0.05, with 24 (92%) showing strict ordering of mean expression levels with SCLC > NSCLC > HBEC. Of the 15 miRNAs that are under-expressed in SCLC cell lines relative to non-SCLCs, 14 (93%) show ordered expression at a significance level of 0.05, with 10 (66%) showing strict ordering of mean expression levels with SCLC < NSCLC < HBEC. These results suggest that expression of a set of miRNA changes progressively from normal cells to NSCLC tumor cells to SCLC tumor cells.

**Table 2:** Sequential changes in expression of individual miRNAs that are differentially expressed between the cell line groups

miRNA	SCLC	NSCLC	HBEC	SCLC NSCLC	SCLC HBEC	NSCLC HBEC	p SCLC NSCLC	p SCLC HBEC	p NSCLC HBEC	pJT	Location
Over-expressed in SCLC cell lines											
miR-338	0.47 ± 0.16	0.08 ± 0.04	0.01 ± 0.00	5.71	47.33	8.29	1.62E-03	8.08E-03	2.38E-01	1.99E-05	17q25.3
miR-101	2.46 ± 1.10	0.52 ± 0.25	0.25 ± 0.08	4.72	9.72	2.06	5.22E-03	3.50E-02	4.20E-01	6.41E-05	1p31.3,9p24.1
miR-98	1.79 ± 0.86	0.51 ± 0.27	0.62 ± 0.11	3.52	2.91	0.83	1.56E-02	1.12E-01	7.49E-01	8.96E-03	Xp11.22
miR-106b	0.47 ± 0.20	0.15 ± 0.08	0.07 ± 0.01	3.26	6.78	2.08	1.03E-02	3.41E-02	4.20E-01	3.31E-05	7q22.1
miR-17-5p	1.07 ± 0.57	0.33 ± 0.19	0.29 ± 0.07	3.25	3.72	1.15	2.95E-02	1.12E-01	8.56E-01	9.49E-04	13q31.3
miR-106a	1.26 ± 0.59	0.41 ± 0.23	0.31 ± 0.05	3.10	4.06	1.31	1.96E-02	7.11E-02	7.39E-01	6.25E-04	Xq26.2
miR-96	0.73 ± 0.28	0.26 ± 0.10	0.12 ± 0.05	2.77	6.24	2.25	1.03E-02	3.14E-02	3.36E-01	4.62E-05	7q32.2
miR-15a	0.45 ± 0.15	0.17 ± 0.04	0.18 ± 0.08	2.63	2.55	0.97	5.12E-03	5.48E-02	9.39E-01	3.49E-03	13q14.3
miR-92	0.44 ± 0.17	0.17 ± 0.08	0.15 ± 0.04	2.54	2.96	1.16	1.33E-02	5.48E-02	7.91E-01	5.42E-04	Xq26.2
miR-326	0.49 ± 0.20	0.20 ± 0.11	0.05 ± 0.01	2.49	10.45	4.19	2.45E-02	2.71E-02	3.36E-01	1.04E-04	11q13.4
miR-1	0.09 ± 0.03	0.04 ± 0.03	0.01 ± 0.01	2.40	6.42	2.68	3.92E-02	2.71E-02	5.04E-01	1.24E-03	20q13.33,18q11.2
miR-15b	0.63 ± 0.24	0.26 ± 0.09	0.23 ± 0.10	2.39	2.78	1.17	1.56E-02	7.07E-02	7.75E-01	2.72E-03	3q26.1
miR-195	2.74 ± 1.23	1.19 ± 0.45	0.60 ± 0.06	2.30	4.55	1.98	3.51E-02	5.48E-02	3.36E-01	4.06E-04	17p13.1
miR-103	0.91 ± 0.26	0.41 ± 0.11	0.29 ± 0.07	2.23	3.16	1.42	5.12E-03	1.99E-02	4.20E-01	7.54E-05	5q35.1,20p13
miR-135	0.28 ± 0.12	0.13 ± 0.03	0.08 ± 0.02	2.19	3.41	1.56	2.95E-02	6.50E-02	3.36E-01	2.25E-04	3p21.1,12q23.1
miR-301	0.74 ± 0.28	0.35 ± 0.44	0.05 ± 0.02	2.12	15.95	7.53	1.14E-01	1.68E-02	5.04E-01	2.72E-03	17q22,22q11.21
miR-328	0.76 ± 0.31	0.36 ± 0.19	0.04 ± 0.03	2.12	19.06	9.00	4.42E-02	2.24E-02	2.38E-01	1.42E-04	16q22.1
miR-93	0.94 ± 0.38	0.45 ± 0.09	0.42 ± 0.13	2.07	2.23	1.07	2.95E-02	1.12E-01	7.94E-01	8.27E-04	7q22.1
miR-16	1.04 ± 0.40	0.51 ± 0.15	0.33 ± 0.10	2.03	3.14	1.55	2.95E-02	5.48E-02	4.20E-01	5.42E-04	13q14.3,3q26.1
miR-324-5p	0.43 ± 0.16	0.22 ± 0.22	0.09 ± 0.03	1.95	4.80	2.46	1.14E-01	3.18E-02	5.93E-01	1.24E-03	17p13.1
miR-107	0.71 ± 0.13	0.38 ± 0.13	0.27 ± 0.09	1.86	2.62	1.41	4.74E-03	4.78E-03	4.64E-01	1.66E-04	10q23.31
miR-149	0.24 ± 0.08	0.15 ± 0.12	0.07 ± 0.03	1.56	3.58	2.29	2.12E-01	3.18E-02	4.99E-01	5.02E-03	2q37.3
miR-181c	0.39 ± 0.12	0.25 ± 0.12	0.13 ± 0.07	1.52	2.91	1.91	1.14E-01	3.20E-02	4.26E-01	4.45E-03	19p13.12
miR-148b	0.24 ± 0.10	0.17 ± 0.11	0.06 ± 0.04	1.39	4.24	3.05	3.38E-01	4.69E-02	4.20E-01	5.00E-02	12q13.13
miR-142-3p	0.13 ± 0.05	0.10 ± 0.07	0.03 ± 0.02	1.31	4.03	3.09	4.11E-01	4.46E-02	4.20E-01	1.72E-02	17q22
miR-30c	2.97 ± 0.87	2.47 ± 1.34	1.12 ± 0.09	1.20	2.65	2.20	4.72E-01	3.18E-02	4.20E-01	5.00E-02	1p34.2,6q13
Under-expressed in SCLC cell lines											
miR-199a*	0.16 ± 0.11	0.28 ± 0.28	0.74 ± 0.18	0.56	0.21	0.37	3.72E-01	1.43E-03	2.73E-01	2.11E-02	19p13.2,1q24.3
miR-27a	0.31 ± 0.23	0.74 ± 0.27	0.90 ± 0.10	0.42	0.35	0.83	2.73E-02	1.86E-02	6.07E-01	1.42E-03	19p13.12
miR-23b	1.86 ± 0.79	5.29 ± 1.55	5.89 ± 0.65	0.35	0.32	0.90	1.99E-03	4.21E-04	7.49E-01	1.93E-04	9q22.32
miR-222	0.47 ± 0.46	1.33 ± 1.07	2.40 ± 0.67	0.35	0.19	0.55	1.14E-01	3.23E-03	4.26E-01	1.09E-03	Xp11.3
miR-221	0.77 ± 0.83	2.19 ± 1.44	3.51 ± 1.17	0.35	0.22	0.62	9.69E-02	1.10E-02	4.64E-01	6.25E-04	Xp11.3
miR-99a	0.28 ± 0.19	0.85 ± 0.70	0.93 ± 0.20	0.33	0.30	0.91	1.03E-01	5.64E-03	9.25E-01	8.00E-03	21q21.1
miR-24	0.62 ± 0.39	1.93 ± 0.70	2.05 ± 0.12	0.32	0.30	0.94	5.12E-03	2.80E-03	8.76E-01	8.27E-04	9q22.32,19p13.12
miR-29c	0.56 ± 0.20	1.97 ± 1.13	1.59 ± 0.71	0.29	0.36	1.24	1.75E-02	1.68E-02	7.86E-01	1.25E-02	1q32.2
miR-23a	1.19 ± 0.74	4.32 ± 1.83	5.76 ± 0.96	0.27	0.21	0.75	5.12E-03	2.50E-04	4.97E-01	4.70E-04	19p13.12
miR-205	0.45 ± 0.15	1.86 ± 3.04	18.38 ± 4.63	0.24	0.02	0.10	3.03E-01	9.67E-06	9.08E-03	1.79E-01	1q32.2
miR-29b	0.72 ± 0.33	3.07 ± 1.49	2.31 ± 1.38	0.23	0.31	1.33	5.22E-03	3.18E-02	7.14E-01	8.00E-03	7q32.3,1q32.2
miR-29a	0.75 ± 0.29	4.08 ± 2.53	3.73 ± 1.63	0.18	0.20	1.09	1.27E-02	3.23E-03	9.07E-01	6.36E-03	7q32.3
miR-22	0.05 ± 0.02	0.33 ± 0.07	0.24 ± 0.12	0.16	0.22	1.39	3.55E-06	5.64E-03	4.26E-01	1.85E-03	17p13.3
miR-21	4.35 ± 6.37	27.93 ± 10.26	11.01 ± 4.60	0.16	0.39	2.54	1.99E-03	2.23E-01	2.73E-01	1.91E-02	17q23.1
miR-31	0.04 ± 0.06	0.64 ± 0.39	5.65 ± 0.96	0.07	0.01	0.11	5.22E-03	3.85E-07	2.34E-04	2.25E-04	9p21.3

Shown are the mean expression level of each miRNA in SCLC, NSCLC, and HBEC cell lines, p values from two-sided t-tests comparing expression for each miRNA between the three groups, corrected for multiple comparisons using the Benjamini and Hochberg FDR method [27], and p values from the Jonckheere-Terpstra test for ordered alternatives (pJT). The last column contains the chromosomal location.

## Discussion

miRNAs have been intensively investigated as diagnostic markers in various cancers and cancer subtypes [5,6,30]. However, few studies have specifically investigated the diagnostic value of miRNA profiles in SCLCs. In this study, we show that more miRNAs are differentially expressed between SCLC cell lines and HBECs than between NSCLC cell lines and HBECs; only two miRNAs were significantly differentially expressed between the NSCLCs and HBEC cell lines. The similarity between the HBEC and NSCLC miRNA profiles reflects the close histological relationship between HBECs and NSCLCs [31-33]. On the other hand, the distinctive miRNA expression signature of SCLCs as compared with NSCLCs and HBECs suggests the possibility of developing miRNA profiling as a diagnostic tool for distinguishing SCLCs from NSCLCs and normal lung tissues. The development of miRNA profiling as a diagnostic tool could potentially benefit SCLC diagnosis from two perspectives. First, miRNA profiling would add a more quantitative aspect to the diagnosis of SCLC. Although the SCLCs share common genetic abnormalities and histological features and represent the most aggressive subtype of lung cancer in general, the survival and prognosis of SCLC patients diagnosed at the same stage vary [34,35], suggesting that quantitative molecular traits are related to the degree of malignancy. However, current diagnosis of SCLC is primarily determined histologically [36], which is not sufficient to quantitatively evaluate malignancy and prognosis. Several studies have shown that miRNA expression levels are related to cancer prognosis [37-40]. Similarly, the quantification of aberrant expression levels of miRNAs in SCLCs may serve as a reliable tool for the prediction of SCLC prognosis. Second, the miRNAs identified as over-expressed in SCLCs may serve as early and non-invasive detection markers. Recent findings have shown that miRNAs are secreted into blood and are detectable in serum, showing potential as non-invasive markers for diseases [41,42]. Inexpensive, non-invasive detection methods are suitable for the development of large-scale screening of high-risk populations and may therefore significantly advance the early diagnosis of cancers. Given the aggressive nature of most SCLCs, the development of highly sensitive and specific non-invasive molecular diagnostics based on miRNA profiling could be of great clinical benefit. Overall, the miRNAs identified as differentially expressed in SCLC compared to NSCLC and normal cells hold promise as early, noninvasive and quantitative markers of SCLCs and warrant further investigation.

Our results suggest that miRNAs may play an important role in the pathogenesis of SCLCs. Although there is evidence to support NSCLCs as originating from HBECs [31-33], the findings on the histological origin of SCLC remain somewhat controversial [43-45]. Previous studies suggest that a transition between NSCLC and SCLC can occur during lung tumor progression and that neuroendocrine differentiation of NSCLCs, which has been postulated to be an intermediate step between NSCLC and SCLC, is related to poor prognosis and early metastasis [46-48]. However, the mechanisms involved in this transition between the two subtypes are not completely understood. Our results show that of the 41 miRNAs that are differentially expressed between the three groups of cell lines, 34 (83%) show a trend of progressive differential expression from HBECs to NSCLCs to SCLCs (Table 2). These results support the hypothesis that differential expression of miRNAs could contribute to the differentiation of lung cancer cells from one subtype to another, in which SCLC could result from NSCLC cells by gradually acquiring SCLC properties through the cumulative dysregulation of miRNAs, and that manipulating the levels of specific miRNAs levels might prevent the differentiation of lung cancer cells toward a more malignant phenotype.

Changes in miRNA expression can lead to tumorigenesis, but the many complex interactions between miRNAs and their targets that occur during these processes are not fully understood. A variety of studies have linked miRNA dysregulation with malignant transformation [49]. However, the role of miRNAs in SCLC pathogenesis has not been extensively studied. Our investigation identified a group of miRNAs that show a progressive differential expression from HBECs to NSCLC and SCLC cells. Several of the miRNAs identified in this study have been shown to be associated with various cancer types in previous studies. For example, we found significant overexpression of miR-103, miR-107, miR-301 and miR-338 in lung cancer cells as compared to HBECs. These miRNAs have been shown to be over-expressed in several types of cancers including lung cancers [17,50,51], and high expression of miR-103 and miR-107 were correlated with poor survival in cancer patients (esophageal squamous and pancreatic tumors) [51,52]. These miRNAs might contribute to common pathways during the transformation of normal cells to tumor cells during lung cancer pathogenesis, and the greater extent of aberrant expression of these miRNAs in SCLCs relative to NSCLCs might contribute to the more aggressive phenotype of the former.

Our study also identified a group of miRNAs that might contribute to the establishment of SCLC features and the specific phenotypes that differentiate SCLC from NSCLC. For example, we found over-expression of miR-17-5p in SCLCs compared to NSCLCs. This miRNA was recently shown to target Rbl2, a member of the Rb family [53]. Rb is a tumor suppressor that induces arrest of the cell cycle at G1 [54]. SCLCs have been shown to exhibit loss of Rb expression in 87-100% of tumors compared to less than 15% in NSCLC [55-57]. SCLC cells were also previously shown to be addicted to continued over-expression of miR-17-5p [58], and forced over-expression of the miRNA cluster that includes miR-17-5p (miR-17-92) was shown to induce embryonic lung epithelial cell proliferation [59]. Coupled with these data, our results suggest that dysregulation of this miRNA could be an important distinction that defines the pathogenesis and phenotypic characteristics of SCLC compared to NSCLC. We also observed a significant increase in miR-135 expression in SCLC cells compared to NSCLC cells. miR-135 has recently been shown to inhibit expression of the tumor suppressor gene Adenomatous Polyposis Coli (APC) in colorectal cancer [60]. Loss of heterozygosity of APC has been shown in both small cell and non-small cell lung cancers, but appears to be more frequent in SCLC [61]. Silencing of this gene by CpG hypermethylation, however, is more frequent in NSCLC compared to SCLC [62], suggesting that various lung tumor subtypes could use different means to down-regulate this tumor suppressor. These findings suggest that SCLC preferentially utilizes microRNA-based regulatory mechanisms to reduce APC expression. miR-29a, -29b and -29c expression was shown be significantly down-regulated in SCLC cells compared to HBECs, whereas these reductions were not seen in NSCLC cells. Expression levels of miR-29a, miR-29b, and miR-29c were previously shown to be inversely correlated with levels of DNA methyltransferase (DNMT) -3A and -3B [14], two key enzymes involved in DNA methylation that have been shown to promote tumorigenesis [63]. Forced expression of these miRNAs also inhibited tumorigenicity *in vitro *and *in vivo *[14]. In SCLC cells, but not NSCLC cells, we also observed significant reductions in miR-24, inhibition of which was previously shown to enhance cell proliferation [64]. These miRNAs might contribute to the specific pathogenesis pathways during the transformation of SCLCs but not NSCLCs.

Several miRNAs identified in our study exhibited expression levels not consistent with previous observations in other cancer types, suggesting contextual dependence of miRNA function in the regulation of tumorigenesis pathways. For example, we observed significantly increased levels of miR-148b in SCLC compared to HBECs; miR-148b has been shown to target DNMT3B [65], with down-regulation of miR-148b observed in metastatic cancers [66]. miR-21, miR-221 and miR-222, which have been shown to be oncogenic miRNAs and up-regulated in certain lung cancer subtypes [67,68], are significantly down-regulated in SCLC. We speculate that these miRNAs may not be the primary driving force for controlling SCLC cell proliferation and survival. Given the large number of miRNAs that are found aberrantly expressed in SCLCs, it is possible that some of these miRNAs play crucial roles in pathogenesis of SCLC. The oncogenic pathways up-regulated by these miRNAs might lead to feedback up-regulation of certain tumor suppressor miRNAs and down-regulation of certain oncogenic miRNAs. Further studies are certainly needed to address this question. We also observed up-regulation of miR-142-3p in SCLC compared to HBECs, although a previous report showed significant repression of this miRNA in lung adenocarcinomas versus normal tissue [69]. Another study showed down-regulation of this miRNA early in tumor development followed by increased expression at the later stages of lung tumorigenesis [70]. Expression levels of this miRNA could therefore vary both with lung tumor subtype and stage of tumor development. miR-1 has also been shown to be expressed at lower levels in lung cancer cell lines, including both NSCLC and SCLC, than in bronchial epithelial cells [71], whereas our results show significant over-expression of miR-1 in lung cancer cell lines compared to HBECs. However, given the extremely low expression levels observed in both the normal bronchial epithelial cells and lung cancer cells in our study, and in normal lung tissues in other studies [71,72], the aberrant expression of miR-1 in lung cancers relative to normal lung cells needs to be evaluated further.

## Conclusions

In summary, our study raises several interesting questions regarding the role of miRNAs in pathogenesis and diagnosis of SCLC. We addressed the potential of miRNA profiling as a diagnostic tool for distinguishing SCLC from NSCLC and normal lung epithelial cells. In addition, our study revealed for the first time that the group of miRNAs that are differentially expressed between lung cancer cell lines and normal lung epithelial cells shows a trend from HBECs to NSCLC cells to SCLC cells, suggesting that increased dysregulation of miRNA expression might be involved in the progression of lung tumors toward a more malignant subtype. Further study on a larger scale is certainly needed to fully define the potential of miRNAs as diagnostic markers of SCLC, as well as the role of specific miRNAs in the pathogenesis of SCLC.

## Abbreviations

SCLC: small cell lung carcinoma; NSCLC: non-small cell lung carcinoma; HBEC: human bronchial epithelial cell.

## Competing interests

The authors declare that they have no competing interests.

## Authors' contributions

JDM and AFG derived the cell lines, LG isolated the RNA, SMH ran the arrays, and JJS and I performed data analysis. LD and AP designed the study, analyzed the data and wrote the manuscript. All authors read and approved the final manuscript.
